# Angle modulated two-dimensional single cell pulsed-field gel electrophoresis for detecting early symptoms of DNA fragmentation in human sperm nuclei

**DOI:** 10.1038/s41598-024-51509-6

**Published:** 2024-01-08

**Authors:** Satoru Kaneko, Kiyoshi Takamatsu

**Affiliations:** Department of Obstetrics and Gynecology, Ichikawa General Hospital, Tokyo Dental College, 5-11-13 Sugano, Ichikawa, Chiba 272-8513 Japan

**Keywords:** Biological techniques, Biomarkers, Urology

## Abstract

We here developed a novel angle-modulated two-dimensional single cell pulsed-field gel electrophoresis (2D-SCPFGE). Variations in current-application-time and rotation angle generated different alignments of DNA fibers and segments. After the first run, the specimen was turned by 150° (2D-SCPFGE-0–150) to detect naturally occurring the earliest stage of DNA fragmentation or 75° (2D-SCPFGE-0–75) to analyze artificially induced cleavage. The former revealed that a part of long chain fibers remained at the origin and long segments were still tangled in the bundle of elongated fibers after the first run. The latter visualized the dose-dependent cleavage of DNA by EcoR1. Multicycle 2D-SCPFGE was useful for generating 2D-alignments of single nuclear DNA fibers, which is the first step for visualization of single-strand breaks on stretched fibers. To date, many articles have accepted the pathogenetic significances of DNA fragmentation in human sperm for male infertility and congenital anomaly. It is necessary to perform multivariate analyses of not only earliest-stage DNA fragmentation but also other types of damage, including single-strand breaks, in sequential DNA fibers. 2D-SCPFGE is the fundamental tool for understanding single nuclear DNA damages.

## Introduction

Apoptosis plays a crucial role in spermatogenetic quality control^1^. DNA repair capacity of mammalian sperm declines in late spermatogenesis^2^, a fraction of sperm accumulates DNA fragmentation due to double-strand breaks (DSBs). While at present, intracytoplasmic sperm injection (ICSI) is a major fertilization procedure in assisted reproductive technology (ART), many cohort studies have reported DNA fragmentation in human sperm nuclei as a major risk factor for sperm-derived congenital anomalies in ART^3–5^.

Sperm chromatin structure assay (SCSA)^6,7^, sperm chromatin dispersion (SCD) test^8,9^, and comet assay (CA)^10,11^ are commonly used to observe nonspecific sequential DNA damage and obtain clinical data. SCSA employs a simple bisection principle wherein the intercalation of monomeric acridine orange (AO) into double-stranded DNA or the adsorption of oligomeric AO to single-stranded DNA produces green or red fluorescence, respectively^6,7^. In the SCD test, DNA damage is inversely proportional to the area of the violet halo, and the absence of the halo indicates serious damage^8,9^. In CA, DNA damage is estimated based on the amount of granular segments discharged electrophoretically from the origin, the so-called “comet tail”^10,11^.

The critical threshold of DSBs in a nucleus is very low^12,13^. However, the incidence of sperm-derived congenital anomalies is not proportional to the number of DSBs, as a number exceeding the threshold results in fertilization failure or pregnancy loss.

Mono-dimensional single-cell pulsed-field gel electrophoresis (1D-SCPFG) with in-gel tryptic digestion has revealed that human semen contains a heterogeneous population of sperm with various stages of DNA fragmentation. Initially, a few large fibrous segments appear beyond a bundle of elongated long chain fibers, and cleavage proceeds until all DNA fibers are degraded to granular segments^14,15^. This observation demonstrated that the comet tail might be derived from the sperm at the end stage of fragmentation.

To revalidate the principles and quantitative performances of SCSA, SCD, and CA, we first separated human sperm with or without DNA fragmentation^16^. Comparative studies have revealed that SCSA is incapable of detecting DNA damage in human sperm. Although green fluorescence is produced due to AO intercalation into double-stranded DNA, red fluorescence is emitted because of AO adsorption to nucleoproteins rather than single-stranded DNA^17^. Human sperm nucleoproteins are composed of protamine^18^, histone^19^, condensin^20^, and cohesin^20^. SCD and CA extracted nucleoproteins with high salt, it was popular formula to extract nucleoproteins such as protamine^8–11^. In SCD, crystal violet-stainable nucleoproteins produce a violet halo, SCD cannot clearly distinguish the comparative standards^16^. In neutral CA, unextracted nucleoproteins remain fixed to DNA due to lack of in-gel proteolysis while unfixed granular segments form the comet tail^16^. In alkaline CA, naked DNA fibers are degraded to granular segments by 0.3 mol/L NaOH without altering the binding capacities of nucleoproteins, thus preventing migration of the newly generated granular segments^16^. Lack of proteolysis diminishes the detection sensitivities of both neutral and alkaline CA^16^.

The present study developed a novel angle modulated two-dimensional single cell pulsed-field gel electrophoresis (2D-SCPFGE), it revealed unexpected results that 1D-SCPFGE^14,15^ also produced false negative for analysis of the early stage of fragmentation. After 1D-SCPFGE, a fraction of long chain fibers remained at the origin and long segments were tangled in the bundle of elongated fibers. Current-application time and rotation angle variations in 2D-SCPFGE provided various profiles of DNA fibers and segments. The present study first provided direct visualization of naturally occurring and artificially induced DNA fragmentation. It is critical to investigate both DSBs and single-strand breaks (SSBs) for comprehensive understanding of sperm-derived congenital anomalies. Assessing two-dimensional profiles of DNA fibers in a single nucleus is the first step of SSBs analyses, followed by the investigation of various alignments of the fibers.

## Results

Human semen specimens (volume: 4.5 ± 0.4 mL, sperm concentration: 76 ± 16 × 10^6^/mL, motility: 62% ± 2.5%, n = 6) were used to prepare motile sperm. After the swim-up, the parameters were as follows: volume, 1.0 mL; concentration, 7.0 ± 0.9 × 10^6^/mL; and motility, 92% ± 3.8%. The rate of DNA fragmentation immediately after the sperm preparation was examined with 2D-SCPFGE-0–150, and ranged from 11 to 18% with a mean of 15% ± 1.5%. The specimen with the minimum rate of DNA fragmentation (11%; 6.8 × 10^6^ sperm/mL, and 95% motility) was chosen as the test sample and preserved in a liquid storage medium to avoid physical cleavage of DNA due to ice crystals. Unless otherwise stated, this specimen was used for all the experiments.

**Figure 1:**
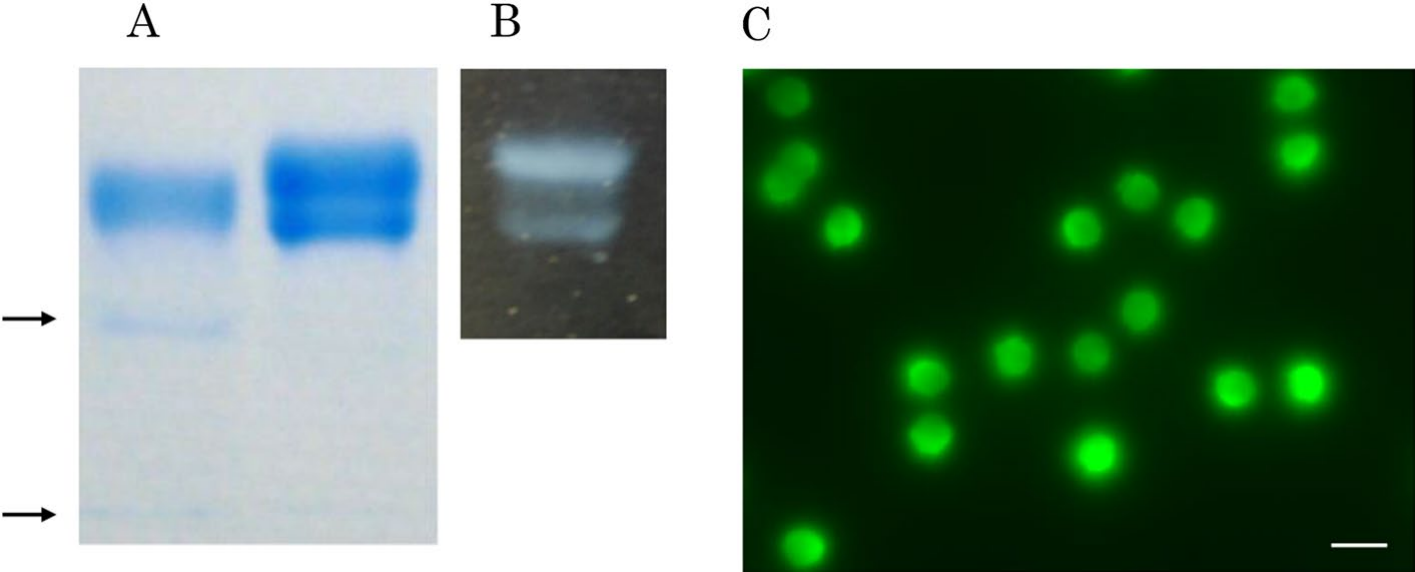
Coextraction of nucleoproteins with proteinase K and SDS allows migration of DNA fibers. (**A**-right) Human sperm extract (10 μL) was electrophoresed and stained with BPB. (**A**-left) The extract (10 μL) was digested with Proteinase K, then electrophoresed, and stained with BPB. The arrows indicate minor bands. (**B**) The extract (10 μL) was electrophoresed and incubated with SDS. The white bands were insoluble complex of protamines and SDS. The full profiles of the electrophoretograms of (**A**) and (**B**) were shown in Supplementary Fig. S1. (**C**) 1D-SCPFGE was performed using the apparatus for 2D-SCPFGE in the same manner as described in “Methods”, however, the sperm was incubated in the modified cell lysis reagent (50 mmol/L Tris–HCl, 1.0 mmol/L EDTA, 5.0 mmol/L DTT, pH 8.2), and electrophoresed for 6.0 min without the angle modulation on the way. After staining with SYBR Gold, the profile was recorded without image enhancement. Scale bars in (**C**) represent 10 µm.

Proteolysis with proteinase K is the standard protocol to extract DNA from somatic cells. However, sperm-specific protamine is resistant to proteinase K. As shown in Fig. 1, two human protamine isoforms were visualized using bromophenol blue (BPB) (Fig. 1A, right) and sodium dodecyl sulfate (SDS) (Fig. 1B) with monosulfate residue as blueish and white agglutinates, respectively. Even after digestion of the extract with proteinase K, one major and two minor fragments retained BPB stainability (Fig. 1A, left). 1D-SCPFGE profile of the test sample after lysis with proteinase K in the absence of SDS suggested that proteinase K did not release DNA fibers (Fig. 1C).

**Figure 2:**
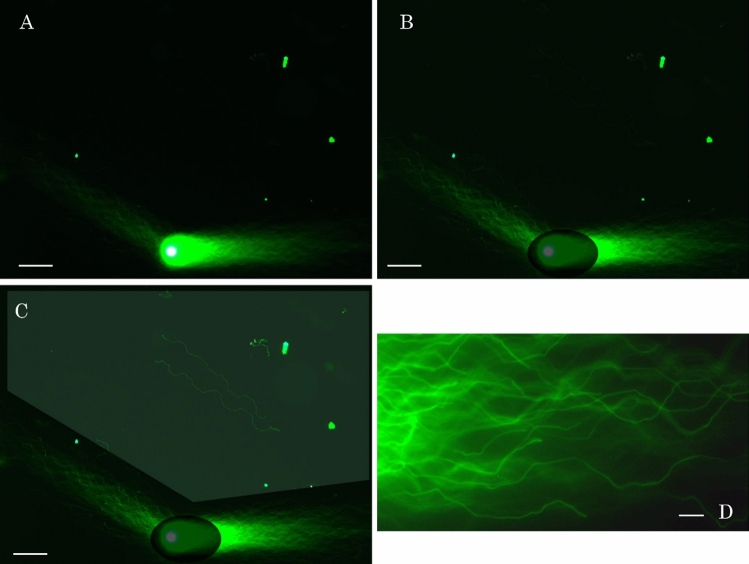
Profiles of 2D-SCPFGE-0–150, setting of the region of interest (ROI), and selective image processing. (**A**) Original fluorescent image of 2D-SCPFGE-0–150, (**B**) the bright area around the origin was enclosed and darkened, then inverted, and brightened by auto-contrast, (**C**) the area at the inner angle of the fan was enclosed polygonally as the region of interest (ROI) and brightened. (**D**) 1000 × magnification image of tips of the fibers after the first run in (**B**). Scale bars in (**A**–**C**) represent 50 µm, in (**D**) represent 10 µm.

**Figure 3:**
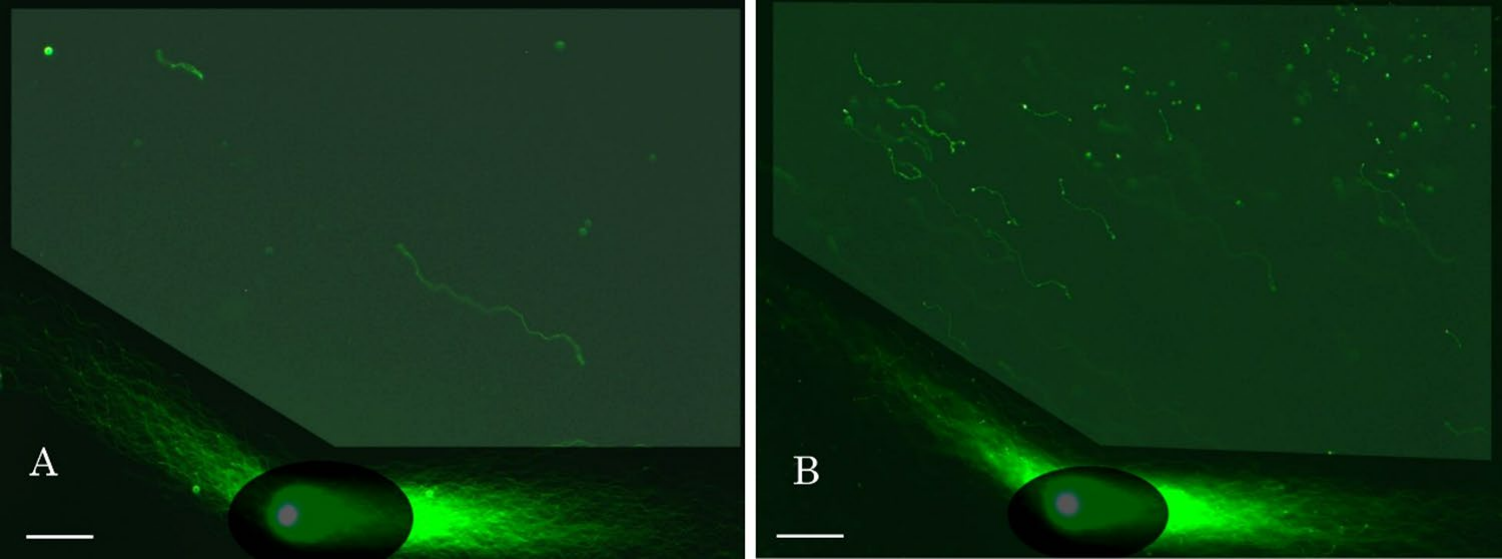
Various sizes of DNA fibers in cryopreserved sperm recorded with 2D-SPFGE-0–150. Scale bars represent 50 µm.

**Figure 4:**
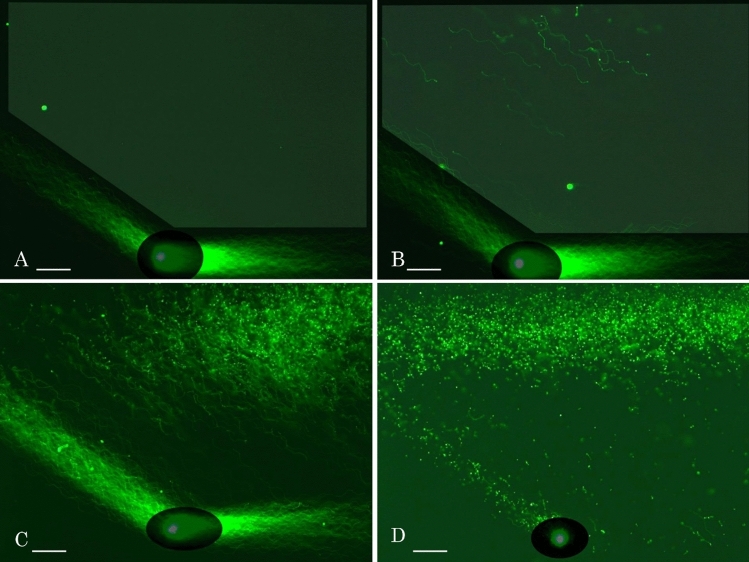
Change in electrophoretic profiles of naturally occurring DNA fragmentation recorded with 2D-SPFGE-0–150. (**A**) Sperm with sequentially intact DNA, (**B**) a dozen long segments in the ROI, (**C**) numbers of fibrous and granular segments in the ROI increased, D: almost all DNA was degraded to granular segments. Scale bars represent 50 µm.

First, we developed 2D-SCPFGE-0–150 (first run: 5.0 min; second run: 3.5 min) to assemble DNA fibers and DNA segments of various sizes in the shooting range of a CCD camera with 200 × magnification. The gel was turned by 150° after the first run, and all the segments previously separated beyond the tips of the elongated long chain fibers were oriented obliquely backward and assembled (Figs. 2, 3, 4). The second run produced an unplanned electrophoretogram, showing newly elongated long chain fibers that were oriented obliquely backward from the origin and spread out in a fan-like shape (Fig. 2A). The fibrous segments including the long segments were drawn out from the bundle of elongated fibers to the inner angle of the fan. Although the elongated long chain fibers and fibrous segments were visible to the human eye under an ocular lens, the high-brightness area around the origin masked their weak fluorescence on digital images (Fig. 2A), and selective image processing was necessary to highlight them. The bright area around the origin was enclosed and darkened, then inverted and brightened by auto-contrast (Fig. 2B). The area at the inner angle of the fan was enclosed polygonally as the region of interest (ROI). Finally, the ROI was brightened by pseudo-exposure corresponding to 4.0s, Consequently, previously unseen two long segments were visualized as shown in Fig. 2C. Figure 2D shows a 1000 × magnification image of tips after the first run in which the fibers changed directions according to the switching interval, following serpentine tracks.

Motile sperm immediately after separation and cryopreserved sperm do not exhibit noticeable differences in electrophoretic features upon analysis by 1D-SCPFGE; however, 2D-SCPFGE-0–150 has improved sensitivity and revealed the unexpected risk of DSBs due to ice crystals. Therefore, we developed a liquid storage medium (50 mmol/L Hepes–NaOH, pH 7.4, 0.5 mmol/L EDTA, 0.1% Triton X-100, 50% propylene glycol) to protect sequence integrity during low-temperature storage. The test sample (rate of DNA fragmentation immediately after preparation: 11%) was preserved at – 20 °C in this medium, with or without propylene glycol, for 1 week. A few (Fig. 3A) to a dozen (Fig. 3B) long segments in the ROI were drawn out from cryopreserved sperm by 2D-SPFGE-0–150. The rate of DNA fragmentation in cryopreserved sperm decreased to 52%. In contrast, the rate of DNA fragmentation (16%) in sperm preserved in the liquid storage medium was similar to that in live sperm. Since 1D-SPFGE cannot detect long segments tangled in the bundle of elongated fibers, physical damage to DNA caused by ice crystals may remain undetected. Therefore, frozen storage is not recommended for quantitation of naturally occurring cleavages.

Figure 4A shows sperm with sequentially intact DNA. The number of fibrous segments in the ROI increased as fragmentation proceeded (Fig. 4B). The same specimen without swim-up was used to observe the advanced stage of fragmentation. The long fibers elongated in two directions, and the numbers of fibrous and granular segments in the ROI increased (Fig. 4C). Finally, almost all DNA was degraded to granular segments, and the size of the origin decreased (Fig. 4D). Thus, 2D-SCPFGE-0–150 not only detected the earliest stage of fragmentation (Figs. 2C, 4B) but also visualized its progression until the end stage (Fig. 4C,D).

**Figure 5:**
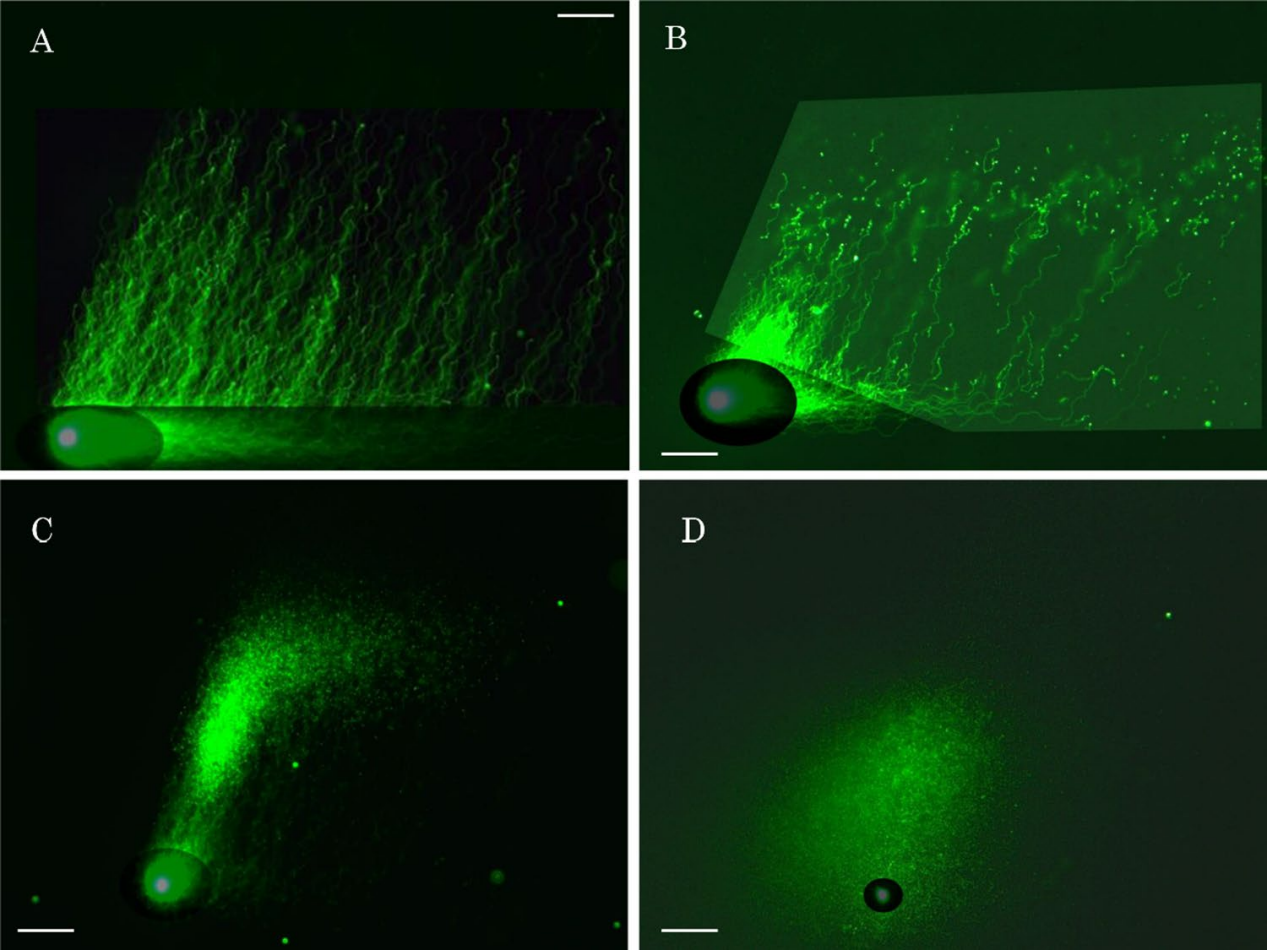
2D-SCPFGE-0–75 profiles of Dose-dependent cleavage of DNA by EcoR1. The concentration of the enzyme was represented on the assumption that it was diffused uniformly in the gel. (**A**) Control, (**B**) 27.7 U/mL, (**C**) 83.3 U/mL, and (**D**) 250 U/mL. Scale bars represent 50 µm.

Figure 5 analyzed the enzymatic cleavage of DNA fibers with EcoR1. The second run in 2D-SPFGE-0–75 (3.0/3.0 min) for DNA cleavage analysis was shortened to 3.0 min to avoid runover of small segments with faster mobility from the shooting range. The fibers were oriented obliquely forward and aligned to each other without overlapping (Fig. 5A, the control). Figure 5B–D show cleavage of DNA by EcoR1 in a dose-dependent manner. In macro-PFGE, in-gel digestion of bacteria by proteinase K needs to be terminated with phenylmethyl-sulphonyl fluoride (PMSF) prior to the addition of restriction endonucleases^21^. Since proteinase K was washed out during the first run, EcoR1 permeated the gel film and cleaved DNA without PMSF treatment. When treated with 27.7 U/mL EcoR1, almost the long fibers elongated after the first run were observed in the ROI as segments of various sizes. Triple-concentrated EcoR1 (83.3 U/mL) digested all long fibers into granular segments, but the tangled fibers around the origin remained. Treatment with 250 U/mL EcoR1 digested the remaining fibers, downsizing the origin and granular segments (Fig. 5D). When using high molecular weight agents for digestion, the permeability of the agent in agarose and the steric hindrance of tangled DNA fibers must be considered.

**Figure 6:**
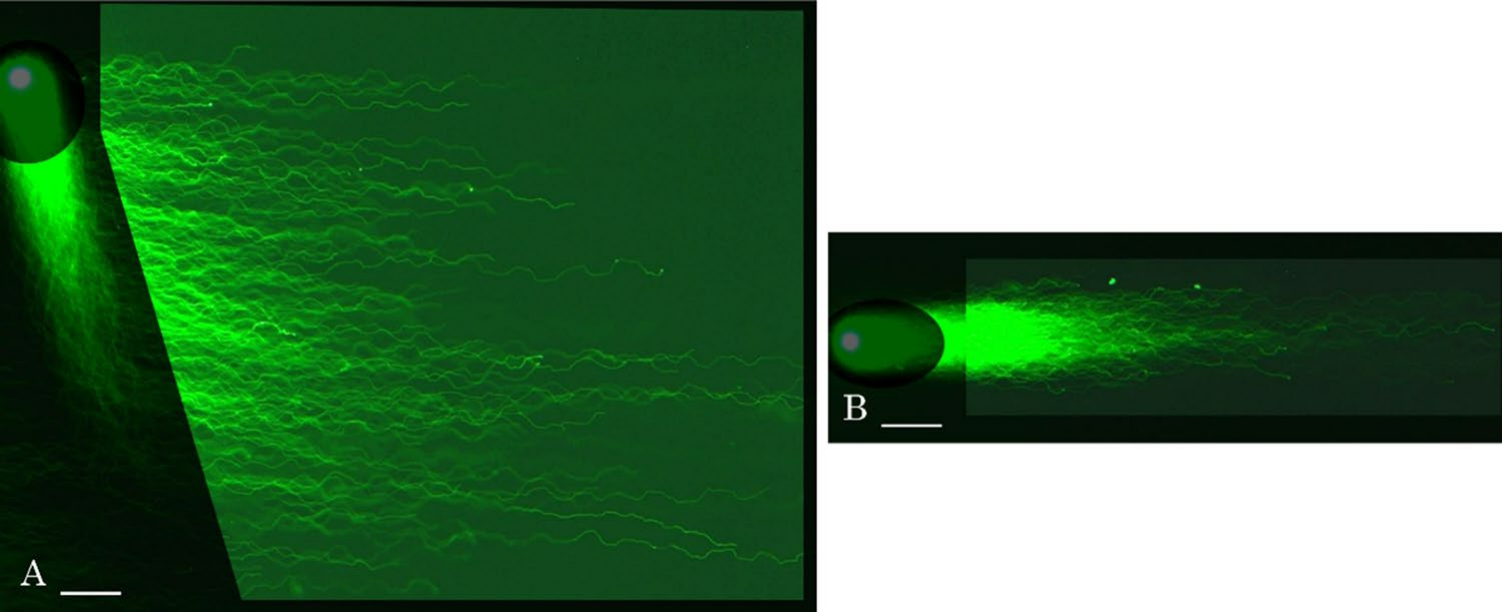
2D-SCPFGE-(-75)–0 to maximize the lengths of the elongated fibers. (**A**) 2D-SCPFGE-(-75)–0 (3.0/10 min), (**B**) 1D-SCPFGE was performed using the apparatus for 2D-SCPFGE in the same manner as described in “Methods”, however, DNA mass was electrophoresed for 10min without the angle modulation on the way. Scale bars represent 50 µm.

Alignment of single nuclear DNA fibers is new approach for single cell analyses, we designed 2D-SCPFGE-(-75)–0 (3.0/10 min) to maximize the lengths of the elongated fibers. The tangled mass of DNA fibers was sleaved by the first run and subsequent change in electrophoretic direction allowed them to elongate laterally and align in parallel without overlapping (Fig. 6A). In contrast, 1D-SCPFGE without the first run drew out markedly fewer and shorter fibers (Fig. 6B). The first run for sleaving the tangled mass of DNA fibers and subsequent change in electrophoretic direction allowed them to elongate laterally and align in parallel without overlapping (Fig. 6A). 2D-SCPFGE with angle modulation is essential to observe early symptoms of DNA fragmentation.

**Figure 7:**
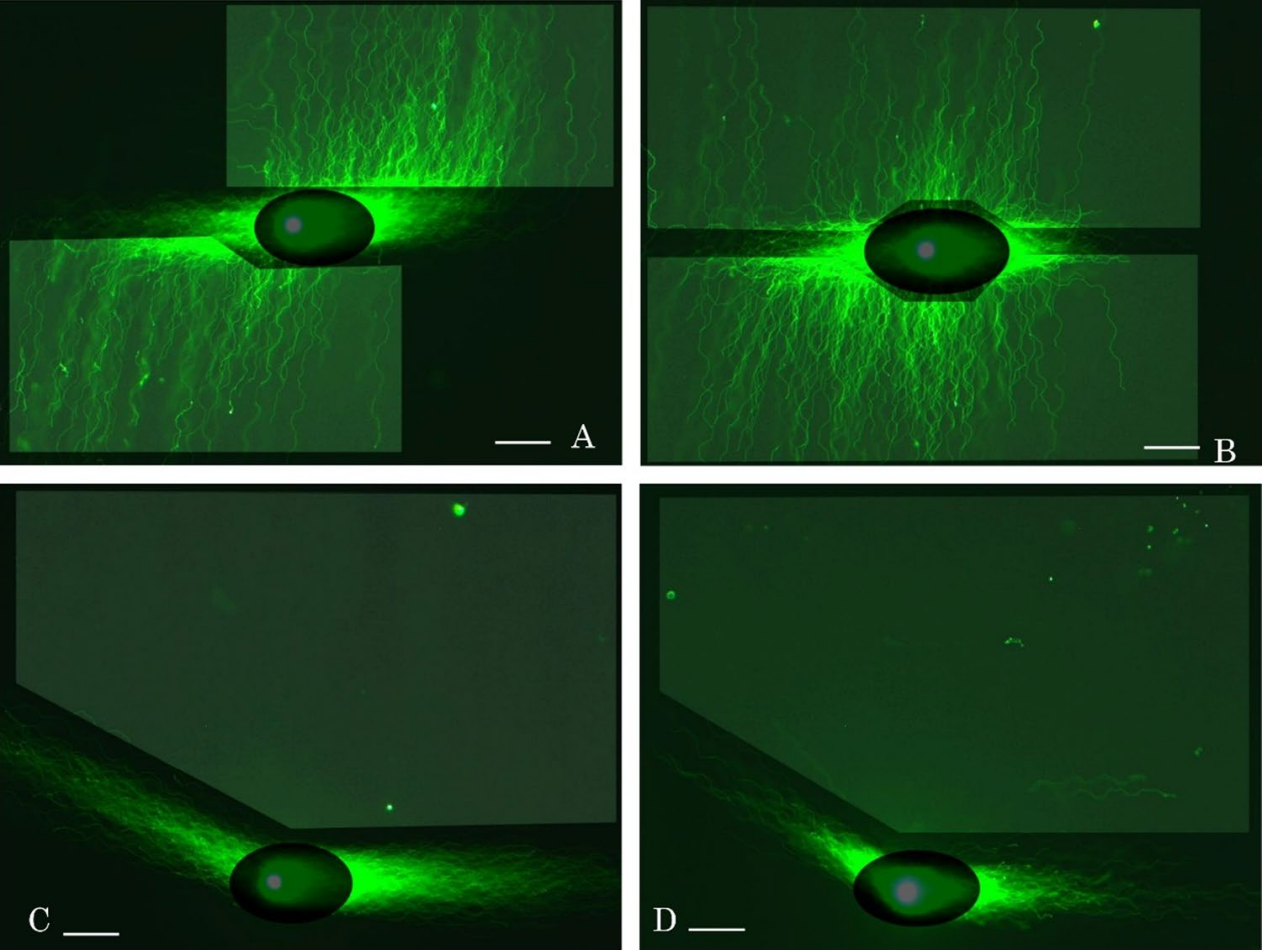
Multicycle 2-D SCPFGE for planer development of DNA fibers. (**A**) 2D-SPFGE-0–90–180–270 (2.0/3.0/2.0/3.0 min), (**B**) 2D-SPFGE-0–180–270–90 (2.0/2.0/3.0/3.0 min), (**C**) even after the third run of SPFGE-0–150–0 (5.0/3.0/3.0 min), there found few changes in the fan-like shape of DNA fibers. Most of sperm gave no segment in the ROI. (**D**) Only one sperm showed lateral migration of two segments which were drawn out from the bundle of DNA fibers elongated at the second run. Scale bars represent 50 µm.

Multicycle rotation is efficient tool for planar development of the fibers. Due to sequential rotations in 2D-SPFGE-0–90–180–270 (2.0/3.0/2.0/3.0 min), the fibers were drawn out laterally from each of the elongated long fibers after the first and third runs (Fig. 7A). In 2D-SPFGE-0–180–270–90 (2.0/2.0/3.0/3.0 min), the fibers initially elongated in counter-directions and thereafter migrated on either side (Fig. 7B). The fiber alignments can be customized for any purpose by the combination of angles and frequency of rotation.

As is evident from Figs. 2, 3, 4, 5, 2D-SPFGE-0–150 was indispensable for detecting long segments. However, to clarify whether the fibers elongated during the second run were still tangled with any segment, we designed 2D-SPFGE-0–150–0 (5.0/3.0/3.0 min). In this approach, the gel was turned around − 150° at the third run. The features of the long fibers after the third run did not differ from those after the second run (Figs. 2, 3, 4, 5, 7C). Of 347 sperm specimens, 296 had no segment in the ROI even after the third run (Fig. 7C), the oblique segments (Figs. 2, 3, 4) were found in 50 sperm, and only one sperm showed lateral migration of two segments, which were drawn out during the third run (Fig. 7D). Although unlikely, 2D-SPFGE-0–150–0 verified the hypothesis, the third run scarcely contributed to improvement of the sensitivity. We recommend the simple 2D-SPFGE-0–150 for the routine analysis.

## Discussion

Bovine pancreatic trypsin (EC.3.4.21.4)^22^, which is used for in-gel digestion of nucleoproteins^14,15^, specifically cleaves lysine and arginine residues. It is inactive at pH 4.7 during gelation and activated at pH 8.2 in cell-lytic medium. Commercially available trypsin is usually contaminated with deoxyribonucleases and readily autolyzed at weak alkalinity. Therefore, trypsin must be purified by strongly acidic treatment and subsequent affinity chromatography^23^. Commercially available proteinase K can be readily used to extract DNA from somatic cells. Proteinase K with chymotrypsin-like substrate specificity is ineffective against protamines (Fig. 1). Since the arginine residues in protamines ionically bond with DNA, competitive extraction with SDS and digestion of other nucleoproteins with proteinase K allows migration of DNA fibers.

Previously, we reported three technical problems of SCSA, SCD, and CA in the analysis of DNA fragmentation in human sperm nuclei^14–16^. First, there is a lack of validation using the comparative standards, leading to inconsistencies regarding the underlying principles. Second, cell samples should be carefully selected because human semen is a heterogeneous population of the sperm at various stages of DNA fragmentation^14^. 1D-SCPFGE suggested that almost all immotile sperm are in the advanced stage of fragmentation^14,17^. However, once fixed and stained or embedded and lysed, it is difficult to determine the type of sperm from which the DNA is derived. Moreover, the test sample is exclusively limited to the separated motile sperm fraction. Third, these analytical methods are subject to clear limits of sensitivity; the critical threshold of DSBs in a sperm is extremely low for complete fetal development^12,13^. The analytical methods need to detect at least a minimum amount of cleavage in a sperm to predict sperm-derived congenital anomalies.

We thought that 1D-SCPFGE fulfills these requirements, the improved sensitivity of 2D-SCPFGE-0–150 revealed further technical issues. Even when pulsed-field impression was used, 1D electrophoresis could not detect physical damage to DNA caused by ice crystals (Fig. 3), yielded false negative results for long segments analysis. The short run for sleaving the tangled mass of DNA fibers and subsequent change in electrophoretic direction were necessary to draw out the long segments from the bundle of fibers (Figs. 2, 3, 4), thus 1D-SCPFGE without the angle modulation could not detect some long fibers cleaved by ice crystals (Fig. 3). 2D-SCPFGE with angle modulation is essential to observe early symptoms of DNA fragmentation, and 2D-SCPFGE-(-75)-0 was optimized to align the fibers without overlapping (Fig. 6A).

The longest segments arise when chromosome 1 is cleaved into halves, however, it remains unclear whether 2D-SCPFGE-0–150 can draw out such long segments. It is necessary to establish several comparative standards with a few to several dozen cleavages in a nucleus to determine the maximum size of detectable segments. A set of size markers is also needed to calibrate the base pairs from images of the segments in micrographs. Until these standards are established, the quantitative performance of 2D-SCPFGE-0–150 will remain undefined. At present, 2D-SCPFGE-0–150 is the most sensitive imaging technique for single nuclear DNA fragmentation (Figs. 2, 3, 4, 5, 6). Percoll density gradient centrifugation with subsequent swim-up is the standard protocol for preparing motile sperm used in clinical ART. The present protocol appended OptiPrep density gradient centrifugation to the conventional protocol to exclude sperm in the end stage of fragmentation^16^. The rate of DNA fragmentation was less than 18% in all the candidates, and the test sample exhibited the minimum rate of 11%. Taking this into consideration, we propose 2D-SCPFGE-0–150 as a reasonably accurate method for analysis of human sperm prepared and stored using current procedures. Furthermore, we suggest as a tentative criterion that when no long segment is visible in the ROI, sperm DNA is sequentially intact.

SSBs are the most common DNA lesions in cells, with tens of thousands induced in each cell everyday^24^. SCSA^6,7^ and alkaline CA^10,11^ are used to investigate SSBs. However, the red fluorescence of AO in SCSA is derived from nucleoproteins, not SSBs^17^. In alkaline CA, the naked DNA fibers are degraded to granular segments by high alkaline, the nucleoproteins remain fixed to newly-generated segments, thus preventing their migration^16^. Detection of SSBs in the sequential DNA is critical to understanding the pathogenetic significance of DNA damage, developing planer profiles of single nuclear DNA fibers by means of 2D-SCPFGE-(-75)–0 (Fig. 6A) and multicycle 2DSCPFGE (Fig. 7A,B) is the first step of SSB analyses.

## Methods

### Ethics statement

Human semen specimens were obtained from volunteers or patients who visited our clinic. The aim of this study and the measurement items were clearly explained to them, and they provided written informed consent" in the manuscript. All the methods in the study have been performed in accordance with the Declaration of Helsinki. The ethical committee of Ichikawa General Hospital approved this study (approval No. 2013-03, -04).

### Discontinuous OptiPrep density gradient centrifugation

Sperm concentration and motility were observed according to the World Health Organization reference manual^25^. Semen specimens were diluted 3 times with 20 mmol/L HEPES–NaOH, Hanks’ solution, pH 7.4, (hereinafter called Hanks). Optiprep (Axis Shield, San Jose, CA, USA) was made isotonic with 20 mmol/L Hepes–NaOH, powder type Hank s’ mixture, 2.0 mg/mL human serum albumin, and pH was adjusted to 7.4; the final concentration was 1.17 g/mL (hereafter referred to as OP). Then the suspension was layered on 0.5 mL OP and centrifuged at 600×*g* for 10 min. The precipitate and interphase layer were resuspended to 1.0 mL. The resuspension was layered on 0.5 mL OP and ultracentrifuged at 10,000×*g* for 10 min to separate the interphase layer from the sediment.

### Differential velocity sedimentation in Percoll and subsequent swim-up

The interphase layer was diluted several times with Hanks, then centrifuged in 90% Percoll (GE Healthcare, Chicago, IL, USA) density gradient as described previously^8^. The 90% Percoll was made isotonic with 20 mmol/L Hepes–NaOH (pH 7.4), Hank’s powder, and 2.0 mg/mL human serum albumin (1.12 g/mL, hereafter referred to as Percoll). Then 5.0 mL Percoll was placed in a conical tip test tube (15mL) and 1.0 mL Hanks was added to it, followed by 10 revolutions at an angle of 30° to create a linear density gradient. The sperm suspension was loaded on the gradient and centrifuged in a swing-out rotor at 400×*g* for 30 min. Hanks (1.0 mL) was added to the precipitate (0.2 mL) and incubated for 60 min at ambient temperature. Motile sperm that swam up into the upper half of Hanks was recovered.

### Human protamine extraction and degradation with proteinase K

Human semen specimens (volume: 3.9 ± 0.3 mL, sperm concentration: 80 ± 5.0 × 10^6^/mL, motility: 71% ± 7.5%, n = 4) were processed as described above, but without swim-up. The precipitates from the Percoll density gradient were combined and condensed to 0.2 mL (sperm: 80 × 10^6^) and mixed with an equal volume of the lytic medium (0.1 M Na_2_CO_3_-NaHCO_3_, 2.0 mol/L guanidine sulfate, 5.0 mmol/L dithiothreitol (DTT), pH 10.0). The gelated DNA was shrunk with 0.4 mL of 10% benzalkonium chloride, then removed using tweezers. The supernatant was mixed with an equal volume of 95% ethanol, and the precipitate was rinsed with 50% ethanol to remove the detergent, followed by resolution in a 0.1 mL 0.05 mol/L Tris–HCl, 20% sucrose, 5.0 mmol/L DTT, pH 8.2.

An aliquot (10 μL/well) of the extract was electrophoresed in acid polyacrylamide gel electrophoresis (PAGE, 18% polyacrylamide gel) according to the common protocol^26^. The gel was stained with 0.05% bromophenol blue (BPB) or 0.1% sodium dodecyl sulphate (SDS) in 0.1 mol/L Na_2_CO_3_-NaHCO_3_, pH 10.0, for 60 min. The extract (10 μL) was digested with 2.0 μg Proteinase K (recombinant grade, 20mg/mL (600 U/mL), Thermo Scientific, MA, USA) in 0.1 mol/L Tris–HCl and 5.0 mmol/L DTT solution, pH 8.2, at 37 °C for 20 min. After electrophoresis, the gel was stained with BPB.

### Two-dimensional single-cell pulsed-field gel electrophoresis

Human sperm (2 × 10^2^) were adhered to MAS-coated glass slides (Matsunami, Tokyo, Japan) by centrifugal auto-smear (Cyto-Tek, SAKURA, Tokyo, Japan). They were embedded in 50 μL of molten 0.36% agarose (50 mmol/L acetate-Na buffer, pH 4.7, 200 μg/mL Proteinase K, 0.1% Triton X-100) to form a 100-μm thick gel coating. After refrigeration at 4 °C, the gel film was incubated in the cell lysis reagent (50 mmol/L Tris–HCl, 0.1% SDS, 1.0 mmol/L EDTA, 5.0 mmol/L DTT, pH 8.2) at 42 °C for 30 min.

The apparatus for 2D-SCPFGE was equipped with three pairs of electrodes arranged at 45°, and the electrophoresis tank was filled with a 20 mmol/L Tris and 1.2 mmol/L EDTA solution, pH 8.9. Current was applied at 2.5 V/cm with 4.0 s switching intervals in the following sequence: left, center, and right. The process was then repeated in the reverse order. The gel film on the glass slide was loaded horizontally on the rack with a pivot for rotation at the intersection of the currents. The area containing the embedded DNA should be exactly positioned upon the pivot. After the first run, the glass slide was turned by 150° (2D-SCPFGE-0–150) or 75° (2D-SCPFGE-0–75) for the second run.

The staining solution (100 μL, diluted [1 × 10^4^] SYBR gold [Molecular Probes, Eugene, OR, USA] in 4.0% 1,4-diazabicyclo [2.2.2] octane [DABCO]-HCl, 20% isopropanol, pH 8.2) was mounted on the gel for 5 min. The excess dye was removed with blotting paper. The electrophoretic profile was observed under an epifluorescence microscope with a green filter (Axio Imager A1, Carl Zeiss Microimaging, Jena, Germany). Still images were recorded using a high-resolution charge-coupled device camera (AxioCam HRC, Carl Zeiss Microimaging) with 0.4 s exposure.

SYBR Gold is one of the most sensitive fluorescent dyes for double-stranded DNA. The human eye can recognize a single SYBR Gold-dyed DNA fiber under an ocular lens, but the fluorescence intensity is too weak to record digital images with currently available equipment. Brightness was adjusted by pseudo-exposure in Photoshop CS6 (Adobe, San Jose, CA, USA). The bright area around the origin was clipped out and darkened, and the region of interest (ROI) was brightened. The present study presumed that when no segment was visible in the ROI, the sperm DNA was sequentially intact. The rate of DNA fragmentation was determined by direct observation under an ocular lens, and more than 100 sperm were counted.

Artificial cleavage of DNA fibers can be observed using 2D-SCPFGE-0–75. The present study demonstrated the dose-dependent action of EcoR1 (Takara Bio. Inc, Otsu, Japan). The naked DNA fibers were elongated in the first run, the gel film was ejected from the apparatus, covered by reaction mixtures (250, 83.3, and 27.7 U EcoR1/mL in 50 μL of 50 mmol/L Tris–HCl, [pH 8.0], 100 mmol/L NaCl, 10 mmol/L MgCl_2_, and 1.0 mmol/L DTT solution), and incubated at 37 °C for 30 min. Thereafter, the slides were placed on the rotating-rack and turned by 75° for the second run. Lateralized DNA segments were enclosed rectangularly as the ROI and enhanced in a similar manner as in 2D-SCPFGE-0–150.

### Supplementary Information

Supplementary Figure 1.

## Data Availability

The datasets generated during and/or analyzed during the current study are available from the corresponding author on reasonable request.
