# Supplementary material for: Angle modulated two-dimensional single cell pulsed-field gel electrophoresis for detecting early symptoms of DNA fragmentation in human sperm nuclei
Source: Sci Rep. 2024 Jan 8;14:840. doi: 10.1038/s41598-024-51509-6 (PMC10774298; doi:10.1038/s41598-024-51509-6)

Supplementary Figure S1 Full profiles of the electrophoretograms of A and B.

After electrophoresis, the gel was stained with bromophenol blue (Left) and incubated with SDS (Right).


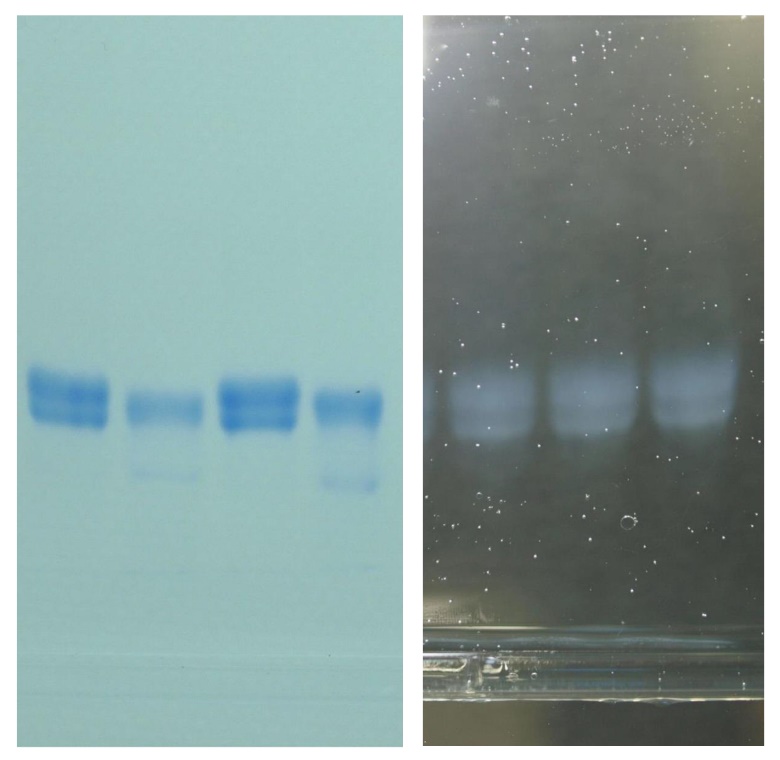

Supplement: Supplementary file 1 — Supplementary Figure 1. [file 41598_2024_51509_MOESM1_ESM.docx]
